# Comparative study of pulpal response following direct pulp capping using synthesized fluorapatite and hydroxyapatite nanoparticles

**DOI:** 10.1186/s12903-024-05285-4

**Published:** 2025-01-04

**Authors:** Eman M. Salem, Omnia M. Abdelfatah, Rania A. Hanafy, Rehab M. El-Sharkawy, Ghadir Elnawawy, Wafaa Yahia Alghonemy

**Affiliations:** 1 Oral Biology Department, Faculty of Dentistry, Pharos University in Alexandria, P.O. Box 37, Sidi Gaber, Alexandria, Egypt; 2 Dental Material Department, Faculty of Dentistry, Pharos University in Alexandria, Sidi Gaber, Alexandria, Egypt; 3 Chemistry Department, Faculty of Dentistry, Pharos University in Alexandria, P.O. Box 37, Sidi Gaber, Alexandria, Egypt; 4 Zoology Department, Faculty of Dentistry, Pharos University in Alexandria, P.O. Box 37, Sidi Gaber, Alexandria, Egypt; 5 Basic Dental Sciences Department, Faculty of Dentistry, Zarqa University, PO Box 2000, Zarqa, 13110 Jordan; 6 Oral Biology Department, Faculty of Dentistry, Tanta University, Alexandria, Egypt

**Keywords:** Direct Pulp Capping, Fluorapatite, Hydroxyapatite, Nanoparticles, Pulp, MTA

## Abstract

**Objective:**

This study aimed to investigate and compare the histological response of rabbit dental pulp after direct pulp capping with 3 different materials: mineral trioxide aggregate (MTA), nanoparticles of fluorapatite (Nano-FA), and nanoparticles of hydroxyapatite (Nano-HA) after 4 and 6-week time intervals.

**Material and methods:**

A total of 72 upper and lower incisor teeth from 18 rabbits were randomly categorized into 3 groups)24 incisors from six rabbits each. MTA Group: teeth were capped with MTA. Nano-FA Group: teeth were capped with fluorapatite nanoparticles. Nano-HA Group: teeth were capped with hydroxyapatite nanoparticles. Blood samples were collected to examine some antioxidant enzymes nitric oxide (NO), superoxide dismutase (SOD), catalase (CAT), glutathione peroxidase (GPx), glutathione (GSH), interleukin-6 (IL-6), and tumor necrosis factor-α (TNF-α). After that, three rabbits from each group were euthanized after 4 and 6 weeks, respectively. Pulp tissues of all teeth in all groups were histologically observed.

**Results:**

The obtained results showed that both Nano-HA induced the formation of thick dentin bridges with irregular dentin patterns at 6 weeks, while MTA and Nano-FA induced no dentin bridge with no tubular dentin pattern. Blood examination at the two intervals revealed no significant increase or decrease in the values of NO, SOD, CAT, GPx, GSH, and TNF-α. However, there was a significant increase in *p*-values of IL-6 in the Nano-FA treated group compared to both MTA and Nano-HA treated groups at the two intervals. Regarding the inflammatory reaction of the dental pulp, the MTA and Nano-HA groups displayed moderate inflammation, followed by Nano-FA, which showed the highest prevalence of nonpathological inflammation. Histological results were consistent with the blood examination. After 4 weeks, the Nano-FA and Nano-HA groups showed pulp fibrosis at the operating site, but the MTA showed only granulation tissues. Plus, dilated blood vessels appeared in the Nano-FA group. After 6 weeks, MTA and Nano-FA groups showed pulp fibrosis at the operating site with the persistence of dilated blood vessels with Nano-FA. The nano-HA group showed dentin bridge formation at the operating site.

**Conclusion:**

MTA and Nano-HA could be considered favorable materials for direct pulp capping, while Nano-FA produces nonpathological inflammatory cell reactions. Moreover, the Nano-HA was the best in dentin bridge formation. Although nano-FA increased the operating site closure, it was noticed that it significantly increased IL-6 compared to MTA at the two intervals and significantly increased IL-6 compared to Nano-HA at 6 weeks, which may be manifested as some nonpathological inflammations in the Nano-FA group compared to the other groups, but it was deemed acceptable to direct pulp capping procedures.

## Highlights

• Nanoparticles stimulate the healing of the pulp after direct pulp capping

• Nano Hydroxyapatite Pulp Capping showed higher results than Nano Fluorapatite

• Nano Hydroxyapatite stimulates dentin bridge formation after 6 weeks

• Nano Fluorapatite Pulp Capping causes nonpathological pulp inflammation

## Introduction

The dental pulp is considered the heart of the tooth as it is responsible for its vitality. Histologically, the dental pulp is a well-vascularized, innervated structure composed mainly of collagen fibers, fibroblast cells, and dental stem cells. The odontoblast layer at the pulp periphery is responsible for dentin formation [1]. Noteworthy, dental pulp damage due to caries or trauma requires an intervention [2]. Millions of teeth are conserved by root canal therapy [3]. Although these current treatment modalities offer high levels of success in many cases, an ideal form of therapy should be biological, non-invasive, or minimally invasive approaches [4]. In this context, various studies are underway to evaluate strategies for pulp regeneration. Despite studies that have promoted signs of full recovery in pilot clinical trials and histological reconstruction, proper regeneration of pulp tissues is still far from being achieved, and the latest advances in functional pulp regeneration are still complex and costly [5–7]. In contrast to research that evaluated tissue engineering-mediated pulp regeneration, which combines stem cells, biomaterials, and growth factors, many other researchers have studied clinically translatable dental materials from a technical and economical point of view to preserve the vitality of dental pulp tissue [8–10]. Procedures known as "vital-pulp treatments" are meant to restore the vitality of pulp tissue that has been severely damaged by trauma, severe dental caries, or iatrogenic infections [11]. Direct pulp capping (DPC) is a less invasive vital pulp therapy designed to heal vital pulp exposure using biocompatible materials [12].

Throughout the past decade, new pulp capping products that showed great potential for success and ease of use have been introduced to facilitate the healing of a damaged pulp [13–15]. Creating a barrier over the pulp which is referred to as a dentine bridge is one of the key functions to avoid early pulp necrosis. Just as crucial as the barrier is the ability to reduce inflammation and infection following the pulp cap placement [9].

Biomaterials functionalized with nanotechnology could be strategic. In nanotechnology, the focus is generally on aspects that become unique when dimensions become small. Using this, nanomaterials for dental pulp can be crucial in providing high surface area for chemical reactions and concentration of many functions in small volumes, thus allowing the efficient use of the same amount of the material [16], in addition to increasing the quality of targeting the injured site while controlling the cost and delivery of the active molecules. Along with the previously listed advantages, Controlling infection and inflammation, as well as coordinating pulp cell colonization and differentiation, are crucial [17].

If the newly introduced nanomaterial showed those favorable characteristics, pulp treatment of primary teeth may undergo a paradigm shift.

Synthetic hydroxyapatite (HA) with a stoichiometric composition as Ca_10_(PO_4_)_6_(OH)_2_ has excellent biocompatibility and it is one of the few materials that is classified as a bioactive material, making it very attractive in several pulp capping procedures to ensure consistent dentin bridge formation. HA, as a pulp capping agent, caused inflammation and necrosis of the pulp in a few cases [18, 19]. Focusing on overcoming the limitations by taking advantage of nanotechnology has led to the development of nano hydroxyapatites (Nano-HA) [20].

Scientific advances in Biomimetic and bio-inspired Nano-HA have been used extensively in the regeneration of skeletal tissue and have provided the basis for the introduction of new technologies into dentistry [18]. Additionally, it was discovered that stem cell adhesion and differentiation are enhanced by the biological characteristics of hydroxyapatite at the nanoscale [21].

The mineral phase of hard tissue contains low but significant amounts of fluorine ions, some of which have been replaced with OH—groups in the apatite structure (Ca_10_(PO_4_)_6_(OH)_2_) that replace OH with F forms fluorapatite Ca_10_(PO_4_)_6_F_2_. Fluorapatite (FA) is characterized by many attractive properties, including bioactivity, biocompatibility, antibacterial properties, high stability, and good hardness values. The first study, which expressed that FA might have better biological properties than HA, was implemented by Kim et al., who concluded that FA has higher biocompatibility than HA [22, 23]. Other research has investigated the effect of different amounts of FA on the behavior of osteoblastic cells and showed that varying the concentrations of FA affected the behavior of osteoblastic cells and had an impact on cellular attachment, proliferation, and differentiation [24]. Theoretically, the biocompatibility of Nano-FA combined with the insights of previous research showed that FA crystal surfaces could induce the differentiation of dental pulp stem cells and mineralized tissue formation [25]. Nano-HA is rich in calcium and phosphate which demonstrated to be useful in increasing dentin regeneration so that reparative dentine can be formed properly. They are able to increase absorption, stability, have good biocompatibility properties to teeth, and contain molecules that have properties identical to human dentin [18]. Previous study by Tredwin et al. [24] showed that the inclusion of fluoride ions and fluoridating HA (Ca_10_(PO_4_)_6_(OH)_2_) to make fluorapatite, can profoundly affect the stability, surface structure, and strength of apatites and it was realized that these changes might introduce desirable effects on osteoblastic cell proliferation. Because the structure of bone and dentin is similar, using fluoro-hydroxyapatites on dentin might help to achieve therapeutic advantages.

To our knowledge, no previous animal or human study has investigated the interaction between Nano-FA on pulp tissue in the case of direct pulp capping. Consequently, the present study aimed to synthesize hydroxyapatite and fluorapatite at the nanoscale level and then assess and compare the pulp response after capping with both nanomaterials.

## Materials and methods

The present investigation was conducted in adherence to the protocols established by the Research Ethics Committee of the Faculty of Dentistry, Pharos University, under ethical code PUA02202407283239.

### Sample size calculation

The minimal sample size is calculated using data based on a previous study [18] aimed to compare the response of exposed dental pulp to mineral trioxide aggregate (MTA), calcium hydroxide, and Nano-HA. The Results on the therapeutic potential of Nano-HA have been promising [18]. In this regard, the material could be considered a substitute and used as a direct pulp capping agent. The sample size was calculated to detect the difference in the modified evaluation criteria used to assess pulpal response. Based on Swarp et al., 2014 [18] results, adopting a power of 80% (β = 0.20) to detect a standardized effect size in the modified evaluation criteria used for the assessment of pulpal response (primary outcome) of 0.480 and a level of significance of 5% (α error accepted = 0.05), the minimum required sample size was found to 24 specimens per group (Total sample size = 72 specimens) [26]. Any specimen loss from the study sample due to processing error will be replaced to maintain the sample size [27].

### Group assignment and animal preparation

Eighteen young male New Zealand white rabbits of 4–5 months old and nearly 1.5–3 kg weight with normal dentition were obtained from the Faculty of Agriculture, Alexandria University. They were selected two weeks before the experiment and examined for any general or dental diseases for exclusion. The animals received food and water and were kept in an environment with controlled lighting and temperature throughout the experimental period, according to the declaration of Helsinki [23].

The procedures were managed under general anesthesia, where 0.4 ml/Kg of atropine sulfate was injected intramuscularly as a premedication drug, followed by an injection of a mixture of ketamine hydrochloride 10% (ketamine alfasan 10%, Woerden, The Netherlands) and xylazine 2% (Adwia, 10th of Ramadan City, Egypt) at a dose of 0.5, and 0.2 ml/Kg body weight, respectively.

A total of 72 upper and lower incisor teeth from 18 rabbits were randomly categorized into 3 groups:

- MTA Group: teeth were mechanically exposed to pulp, and partial pulpotomy was capped with MTA (*n* = 24 incisors from six rabbits).
- Nano-FA group: teeth were mechanically exposed to pulp, and partial pulpotomy was capped with Nano-FA (*n* = 24 incisors from six rabbits).
- Nano-HA group: teeth were mechanically exposed to pulp, and partial pulpotomy was capped with the Nano-HA (*n* = 24 incisors from six rabbits).

## Materials

The chemicals, reagents, and solvents utilized were all analytical reagent grade and were directly employed without additional purification. Double distilled water was consistently utilized in all experiments.

### Synthesis of Nano-FA

The synthesis of Nano-FA powder was carried out via the co-precipitation method [28]. Initially, a solution of calcium nitrate was stirred for 30 min. Subsequently, a second solution containing diammonium hydrogen phosphate (NH_4_)_2_HPO_4_, FW 132.06 g/mol, and assay > 99%) (Merck, Germany) in distilled water was also stirred for the same duration. Ammonium fluoride (NH_4_F, FW 37.04 g/mol, and assay > 99%) (Merck, Germany) was then added to the second solution and stirred for an additional 30 min. The second solution was added drop by drop while adding ammonia ensuring the pH was above 10. After 24 h, solid particles were separated from ammonia using centrifugation. The resulting powder was dried at 70ºC for 24 h in an oven, ground, and stored separately.

### Synthesis of Nano-HA

Nanosized HA samples were prepared via a chemical precipitation approach according to the previously reported protocol [29]. Briefly, the first step in the synthesis of pure Nano-HA powders was the preparation of stock solutions of calcium hydroxide (Oxford Co.) and orthophosphoric acid (Oxford Co.) using distilled water. Then orthophosphoric acid solution was added drop by drop to the calcium hydroxide solution. The experiment was conducted at a temperature of 40°C and a pH of 9.4. After 24 h at room temperature, the powder was washed several times with ammonia (Shanghai Titan Chem Co. Ltd, China) aqueous solution. The product was vacuum-filtered and dried in an oven at 60°C for 24 h. The dried powder was then milled using mortar and pestle and finally calcined at 650°C for 2 h.

### Instrumentation for characterization

Various characterization techniques were employed to analyse the synthesized Nano-FA and Nano-HA in this study. FTIR spectra were recorded throughout the range from 4000 cm^−1^ to 400 cm^−1^ for spectroscopic identification of the functional groups present in Nano-FA and Nano-HA particles. Semi-quantitative analysis of the chemical composition of the prepared Nano-FA and Nano-HA was characterized with energy dispersive x-ray analysis (EDX), it was performed on the powder to estimate its elemental composition. X-ray diffraction analysis (XRD) was applied to determine phase identification and crystallinity. The crystal domain sizes of the prepared Nano-FA and Nano-HA particles were calculated by Scherrer’s equation based on XRD investigations [30]. The morphology and particle size of the produced nanoparticles were observed and analysed by scanning electron microscope (SEM) and high resolution-transmission electron microscopy (HR-TEM). Table 1 provides an overview of the specifics regarding the instrumentation associated with the characterization techniques employed in this research.

**Table 1:** Instruments and their specifications

**Instrument Name**	**Model**	**Data**	**Conditions**	**Technique**
Fourier-Transform Infrared Spectrophotometer (**FT-IR)**	FTIR-3400 s SHIMADZU	FT-IR spectrum	400–4000 cm^−1^	Using KBr pellets
X-ray Diffraction** (XRD)**	SHIMADZU lab × 6100, Kyoto, Japan	XRD spectrum	40 kV, 30 mA, λ = 1 Å, 2θ from 10 to 80, recording steps of the diffraction data of 0.02°, at a time of 0.6 s, at room temperature (25 °C)	X-ray diffractometer, using target Cu-Kα
Scanning Electron Microscope (**SEM)**	JSM-6360LA, JEOL Ltd	SEM images	Imaging mode	Sputtering coating (JEOL-JFC-1100E)
High Resolution-Transmission Electron Microscopy (**HR-TEM)**	JEOL, TEM-2100 plus, Electron Microscope	HR-TEM images	Imaging mode	Thin film coating
Energy Dispersive X-ray **(EDX)**	JSM-lT200, JEOL Ltd	EDX Spectrum	Acceleration voltage 20.00 kV, WD 10.00 mm, Live time 30.00, high vacuum mode	Energy Dispersive X-ray

### Tooth preparation and partial pulpotomy

Each tooth was isolated by a rubber dam, cleaned with pumice, and washed with water. The operative area was disinfected with 2.5% iodine (Aqua Chemicals, Egypt) and 70% alcohol (MediMix, Egypt). A class V cavity with a nearly 1.5 mm to 2 mm dimension was prepared on each tooth's labial surface using a sterile pear-shaped diamond bur (0.10 ISO standards). After cavity preparation, the pulp horn was mechanically exposed to approximately 1 mm in diameter by drilling with a sterile high-speed diamond bur (0.10 ISO standards) under copious sterile water irrigation [18]. After the bleeding was controlled by applying sterile cotton pellets with 0.9% normal saline to the exposed pulp, Nano-FA or Nano-HA was applied to each exposure site in groups Nano-FA or Nano-HA, respectively. The cavity was subsequently lined with resin-modified glass ionomer cement and filled with flowable composite resin (Charisma Diamond One). After the restorative procedure, the rabbits were monitored for any symptoms, and the operative sites were monitored daily. To alleviate pain, subcutaneous injection with a nonsteroidal anti-inflammatory drug at 2 to 4 mg/kg was administered daily for 3 consecutive days.

### Blood sample examination

Blood samples were collected from the tail vein of the rabbits in glass tubes without anticoagulant and left at room temperature for 30 min for spontaneous clotting. Clotted blood was centrifuged at 2000 rpm for 5 min at 4°C pipette off the top yellow serum layer without disturbing the white buffy layer. The serum was frozen at -80°C for analysis, and the serum was used for the determination of the following parameters: some antioxidant enzymes such as nitric oxide (NO), superoxide dismutase (SOD), catalase (CAT), glutathione peroxidase (GPx), glutathione (GSH), interleukin-6 (IL-6), and tumor necrosis factor- α (TNF- α). The total nitric oxide (NO) levels were measured after the conversion of nitrate to nitrite by nitrate reductase (Griess diazotization reaction) using the Nitrite Assay Kit (CAT. No. NO2533, Bio-Diagnostic, Egypt). While the nonenzymatic antioxidant glutathione GSH was measured spectrophotometrically (CAT. No. GR 25 10). TNF-α in tissue was measured using ELISA kits (Cat. No. RK00029) and an Enzyme-linked Immunosorbent Assay (Bioassay technology laboratory (BT lab) in Zhejiang, China (Cat no. E0135Ra) was used for the quantitative detection of IL-6. ELISA kits (Cat. No. RK00029).

### Animal’s euthanization

Three rabbits from each group were euthanized after 4 and 6 weeks, respectively. The rabbit's euthanization was done by intramuscular injection of an overdose of xylazine HCl (30 mg/kg; Adwia, 10th of Ramadan City, Egypt) and ketamine HCl (70 mg/kg; ketamine alfasan10%, Woerden, The Netherlands) and scarified then dissected.

### Histologic assessments

Upper and lower jaws were removed, coded, and fixed into 10% calcium formol (Aqua Med Company, Egypt) for 48 h. After that, they were washed and decalcified through immersion in EDTA 10%. Then, the samples underwent progressively higher alcohol concentrations for dehydration before being conventionally embedded in paraffin. Hematoxylin and eosin (H&E) and Masson Trichrome stains were used to stain 6 μm thick sagittal serial sections of the upper and lower incisor areas (12 upper and lower incisor teeth for each interval in all groups) for histological analysis under a light microscope (Leica ICC50 HD) outfitted with a digital camera, and images of representative regions were captured and annotated [31]. Pulp tissue for each tooth was histologically observed. A single investigator blindly performed the histological analysis.

### Statistical analysis

The data obtained from the blood sample examination was subjected to statistical analysis. Data was fed to the computer and analyzed using IBM SPSS software package version 20.0. (Armonk, NY: IBM Corp) The Kolmogorov–Smirnov test was used to verify the normality of distribution. The tests used were F the test (ANOVA) for normally distributed quantitative variables, to compare between more than two groups, and the post-hoc test (Tukey) for pairwise comparisons. The differences among the groups were expressed as mean ± SD.

## Results

All animals survived the experimental procedures. No gingival or systemic inflammation-related symptoms were noticed.

### Structural characterization of the synthesized Nano-FA

#### FT-IR Spectroscopy

**Fig. 1:**
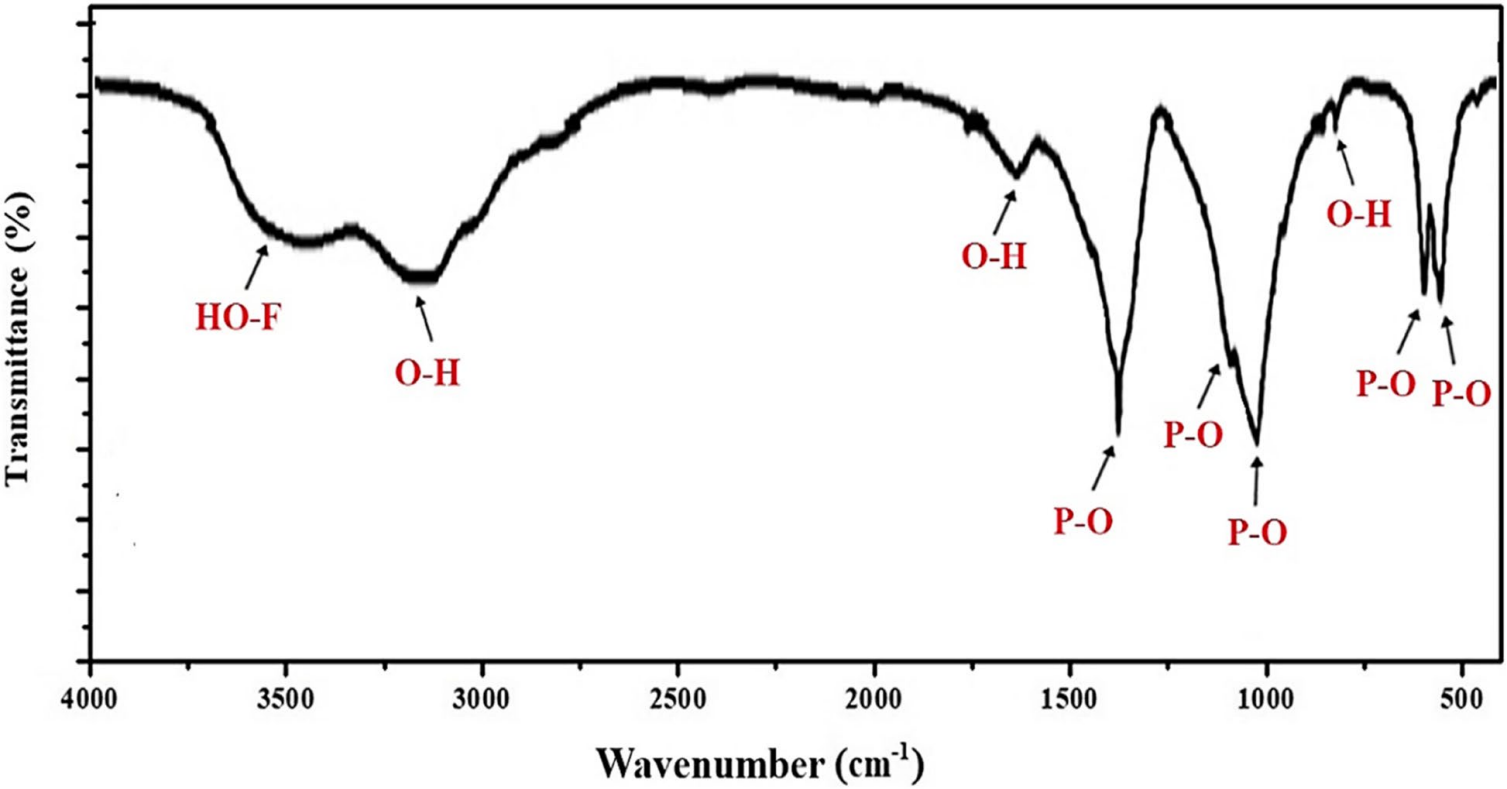
FT-IR spectrum of Nano-FA

Figure 1 displays the FT-IR spectrum of the synthesized Nano-FA within the 4000–400 cm^−1^ range. The vibrations between 1400–900 cm^−1^, associated with the stretching modes of PO_4_, were identified as the distinctive peaks of pure apatite to confirm complete apatite formation. A strong band of PO_4_ was detected at 1031.44 cm^−1^. Subsequent strong bands at 1384 and 564, 604 cm^−1^ are attributed to the v_1_ and v_2_ vibrations of PO_4_, respectively [28]. The FT-IR spectrum of pure FA displays prominent chemical groups such as PO_4_^3−^, OH^−^, CO_3_^2−^, and HPO_4_^2^, indicating the presence of non-stoichiometric fluorapatite [32]. The identification of PO_4_^3−^ groups can be verified by the strong bands detected at 560, 600 cm^−1^, and 1000–1100 cm^−1^. The adsorbed water band exhibits a broad range, from 3600 to 2600 cm^−1^, with a distinct peak at 3157 cm^−1^, and another peak at 3544 cm^−1^. The CO_3_^2−^ group shows weak peaks between 870 and 880 cm^−1^ and a more prominent one at 1450 cm^−1^. It is essential to highlight that the peak between 3536 cm^−1^ and 3545 cm^−1^ corresponds to the OH–F band, as reported in previous studies [33, 34]. Thus, the presence of this peak may suggest the presence of fluoride-substituted HA.

### X-ray diffraction (XRD)

**Fig. 2:**
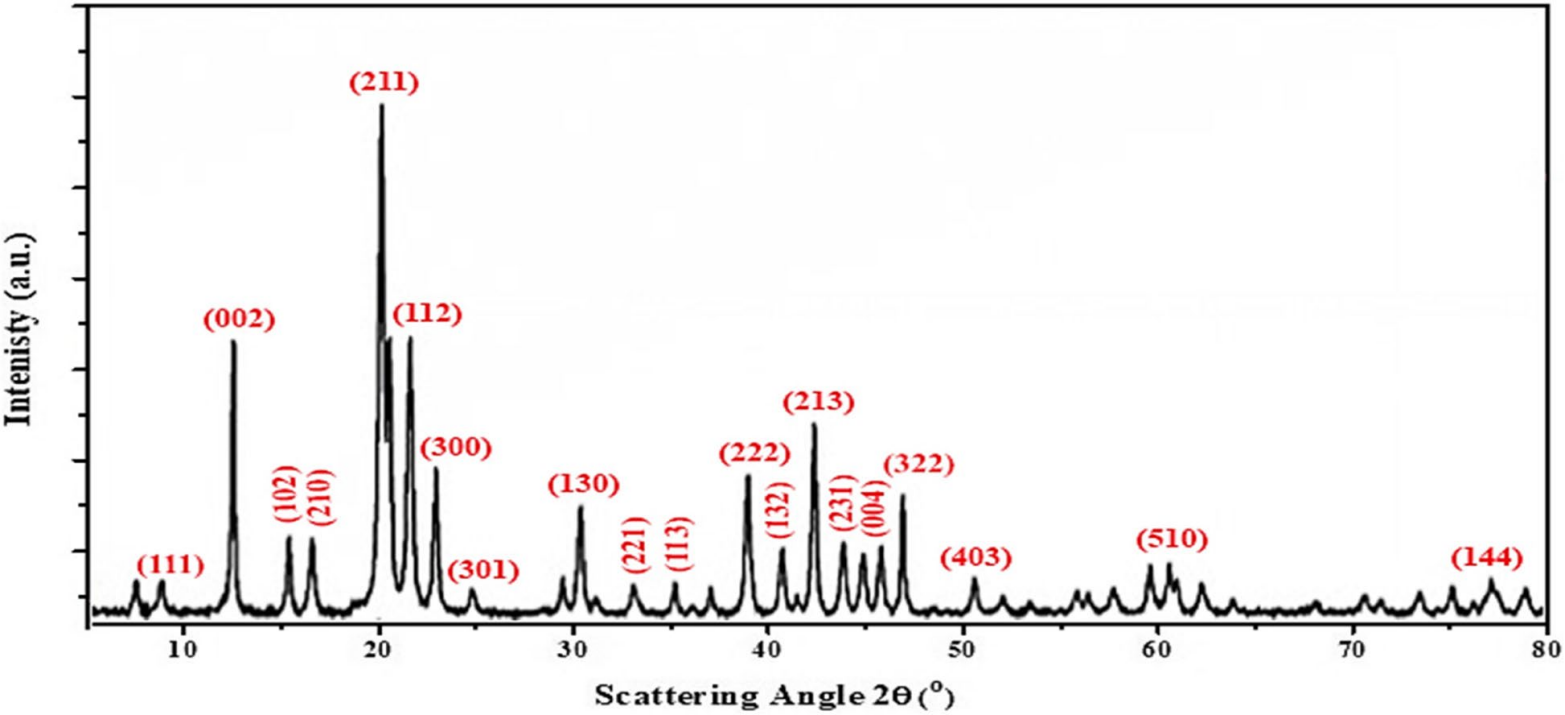
XRD patterns of the prepared Nano-FA

The XRD pattern of the synthesized nano-fluorapatite is shown in Fig. 2. The peak lines representing the FA phase are evident in the figure. Through X-ray powder diffraction measurement Fig. 2, it was verified that the nanocrystalline fluorapatite Ca_5_(PO_4_)_3_F is in a pure crystal phase. The diffractogram obtained was matched against a standard pattern from the Inorganic Crystal Structure Database (No. 9444) [35]. In addition, it is evident from Fig. 2 that, no impurity diffraction peaks, or crystal phases were identified in the XRD patterns of Nano-FA.

### Energy dispersive X-rays (EDX)

**Fig. 3:**
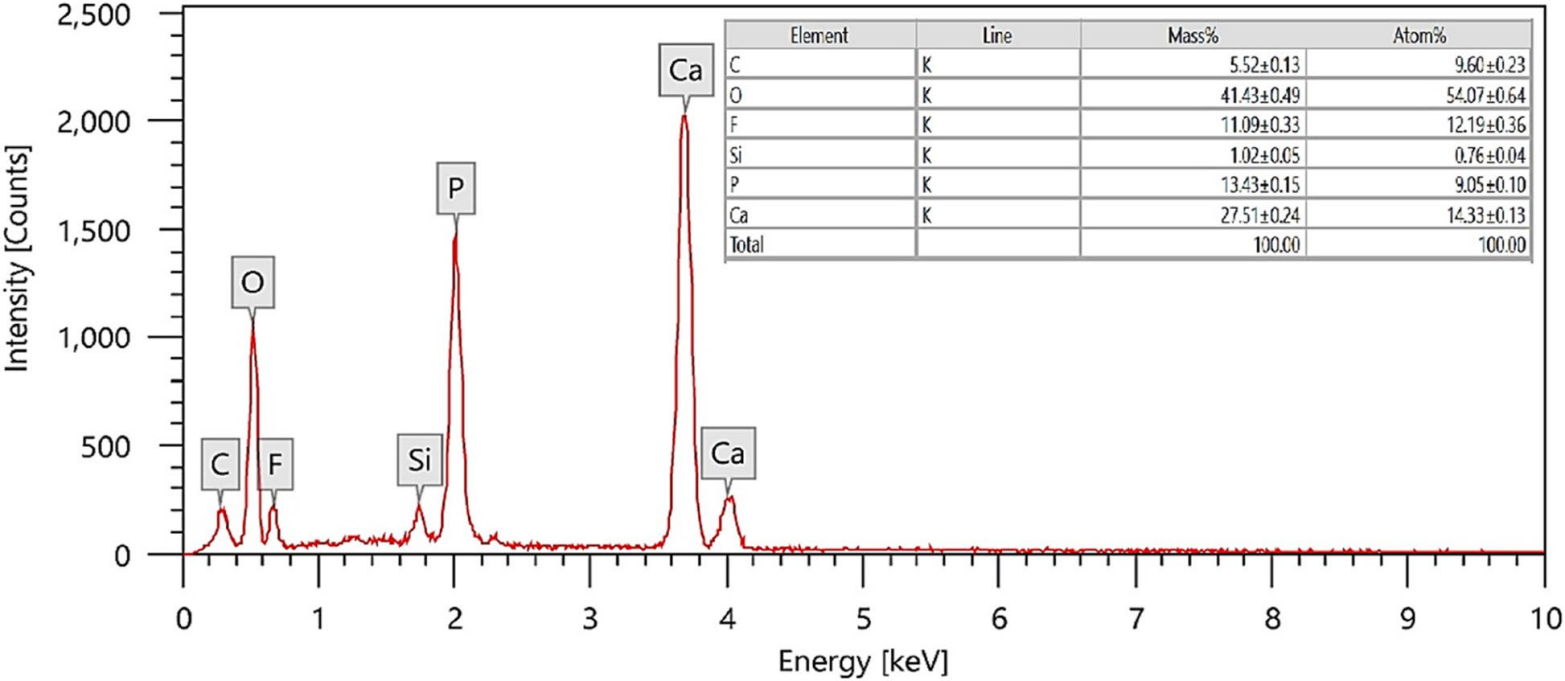
EDX chart of the prepared Nano-FA showing the Ca, P and F content

The EDX technique was used to conduct the elemental analysis of the synthesized Nano-FA sample. Figure 3 illustrates the presence of peaks for O (41.43%), F (11.09%), P (13.43%), and Ca (27.51%), as well as some traces of C and Si impurities. This analysis confirms the successful preparation of Nano-FA powder.

### Scanning electron microscope (HR-TEM) and High-resolution-transmission electron microscope (HR-TEM)

**Fig. 4:**
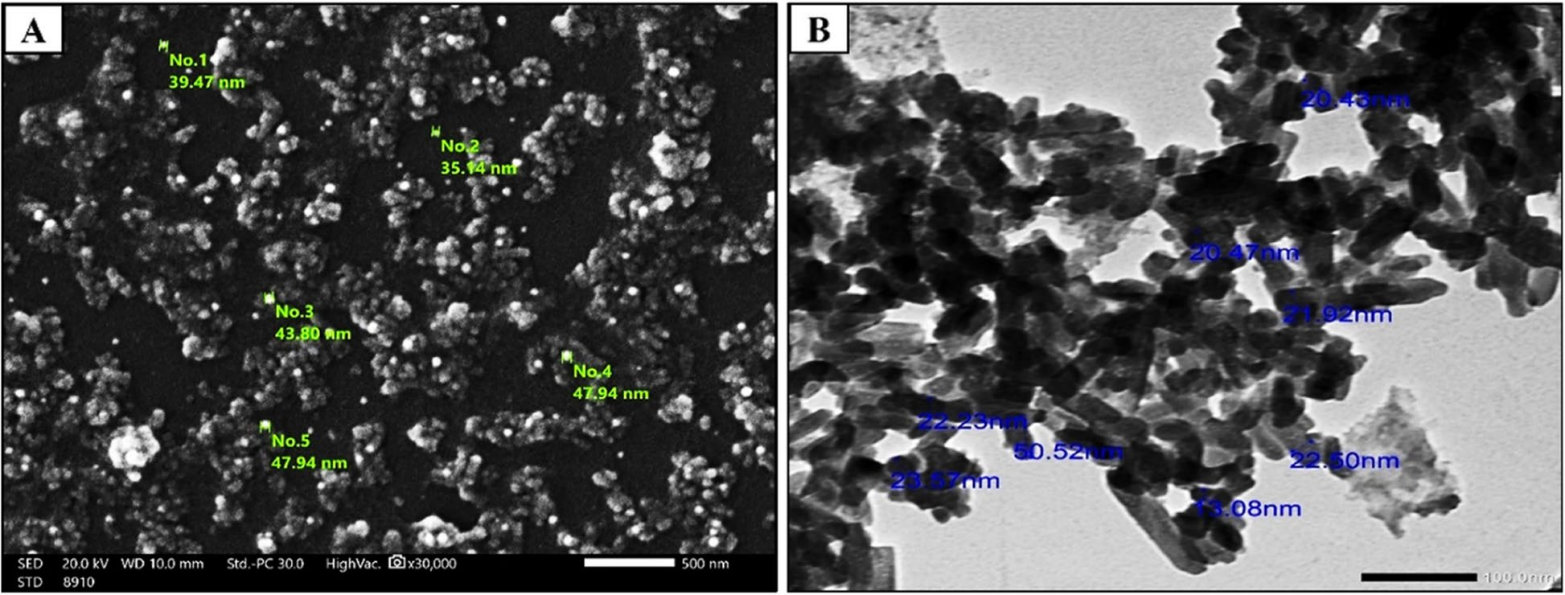
**A** SEM-image at 30000 × magnification showing the surface morphology of Nano-FA, **B** HR-TEM-image at 10000 × magnification showing the semi-spherical shape structure of Nano-FA

The SEM and HR-TEM techniques were employed to analyze the synthesized Nano-FA powder's structure, surface morphology, and particle size. The corresponding images can be found in Fig. 4A and B. The SEM image (Fig. 4A) shows that the Nano-FA exhibited a semi-spherical shape, with a particle size ranging from 35.14 to 47.80 nm. The HR-TEM image **(**Fig. 4B**)** provides similar results, confirming that the Nano-FA particles are also semi-spherical in shape, with an average particle size of 34.34 nm.

### Structural characterization of the synthesized Nano-HA

**Fig. 5:**
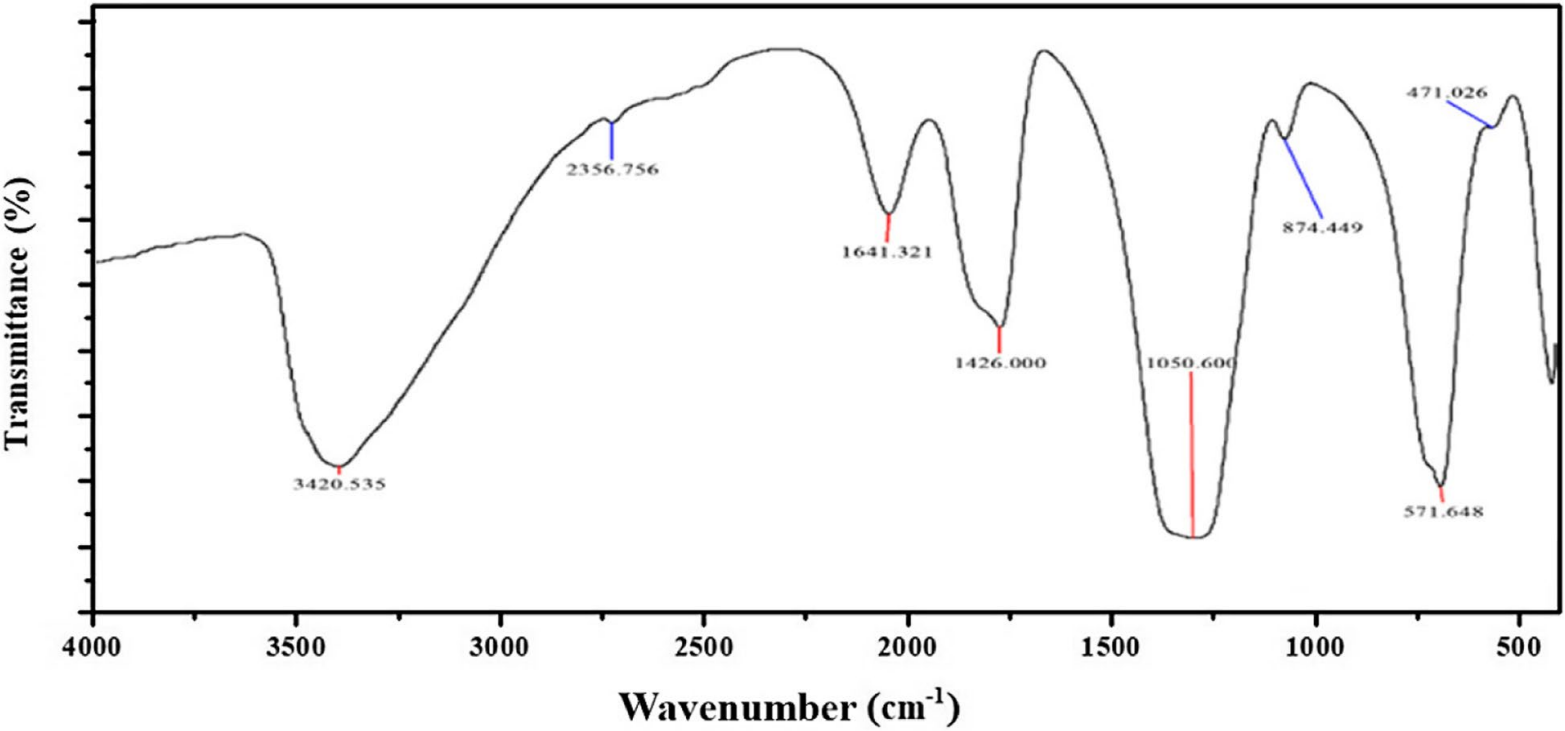
FT-IR spectrum of Nano-HA

**Fig. 6:**
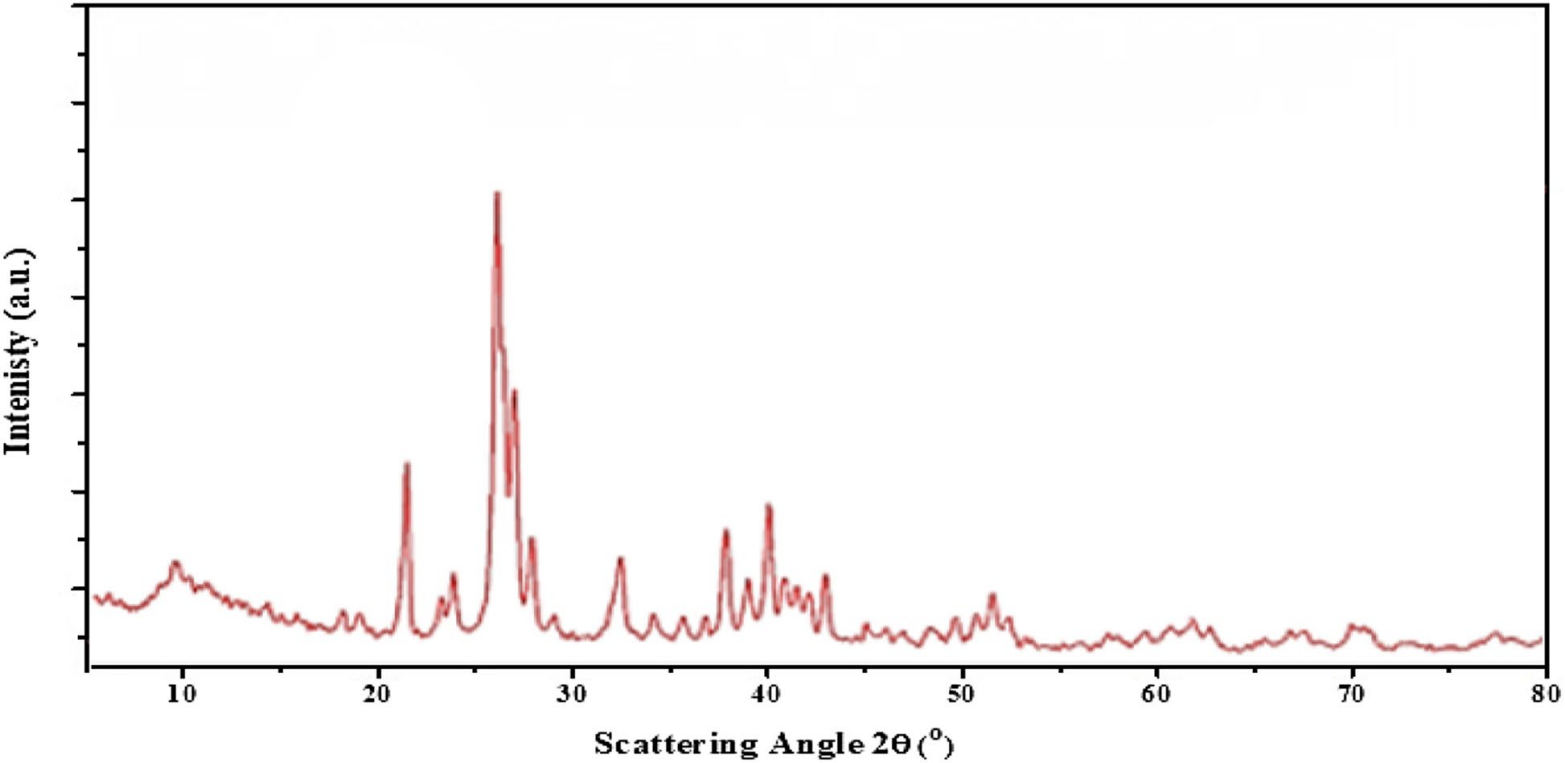
XRD pattern of the prepared Nano-HA

**Fig. 7:**
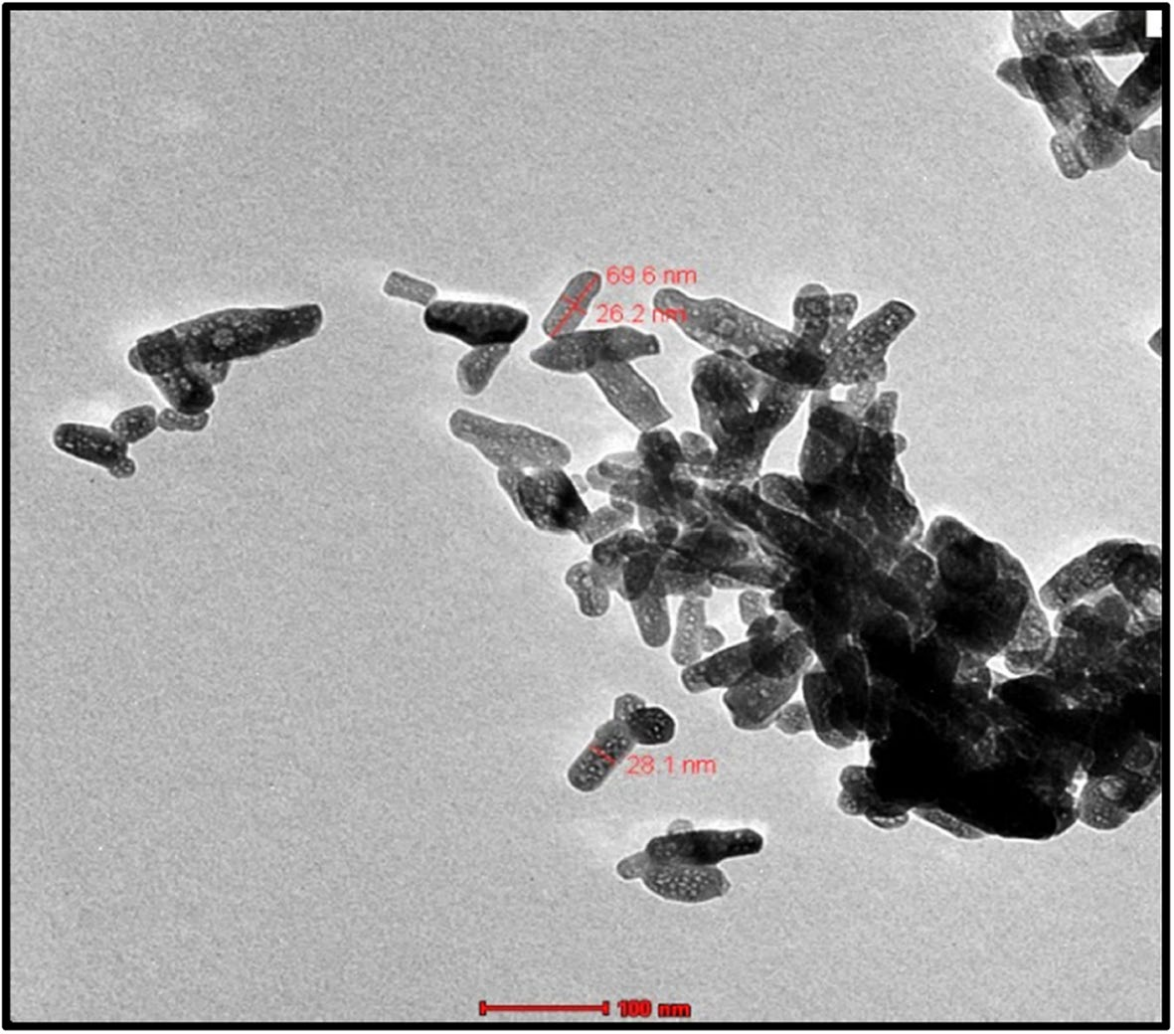
HR-TEM at 10000 × magnification showing the hexagonal rod shape structure of Nano-HA

**Fig. 8:**
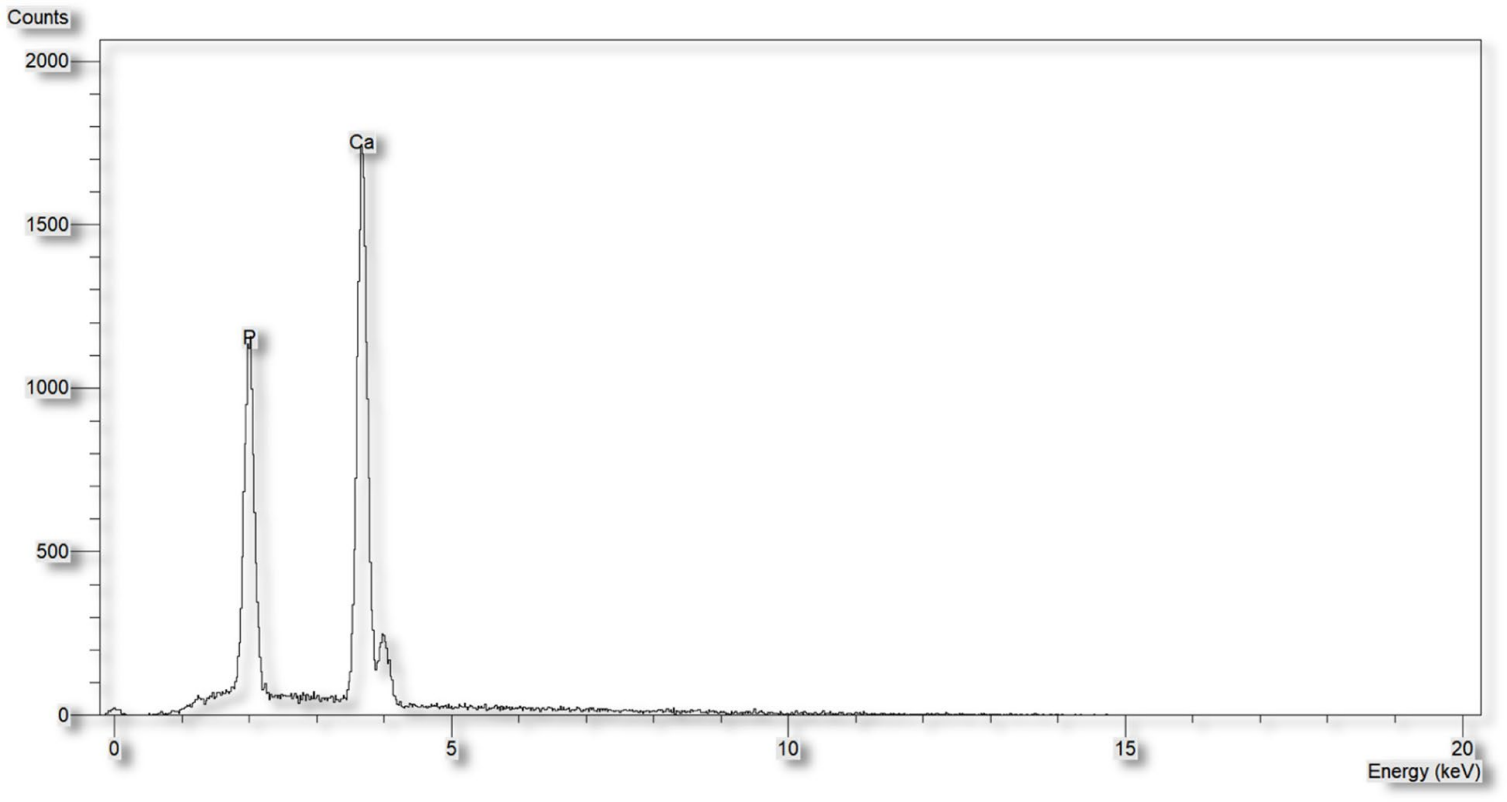
EDX chart of the prepared Nano-HA showing the Ca and P content

The FTIR spectrum analysis of the prepared sample (Fig. 5) showed all the characteristic peaks of pure HA and confirmed the presence of the characteristic vibrations of the phosphate and hydroxyl groups of the Nano-HA, stretching vibration band observed at 3420 cm^−1^ confirms the presence of the hydroxyl group (OH). The peak at 874 cm^−1^ corresponds to a symmetric stretching mode of the P—O bond in the phosphate group also an asymmetric P—O stretching peak was identified approximately at 1050 cm^−1^, which is the most intensified peak among the phosphate group vibration modes. X-ray diffraction (XRD) analysis results (Fig. 6) revealed that the synthesized HA presented characteristic diffraction peaks consistent with JCPDS file no. 09 − 432, indicating a successful synthesis of HA powder. All of the peaks are indexed to a hexagonal lattice of hydroxyapatite crystals. Based on the XRD pattern calculate crystal size by using Scherrer’s equation. The mean crystal size of the prepared Nano-HA particles was found to be 20.4 nm. TEM (Fig. 7) revealed the presence of a hexagonal rod-shaped structure with particle size around 15 to 30 nm in width and 80–100 nm in length. Furthermore, HA particles are not agglomerated and are mono-dispersive. EDX analysis (Fig. 8) determined that the synthesized Nano-HA had a Ca/P ratio estimated as 1.65 which is close to the theoretical value for apatite (1.67) [35].

### Results of blood sample examination

#### Oxidative stress markers and antioxidant enzymes in serum

At 4- and 6-week intervals, the current study revealed no significant increase or decrease in the values of (NO, GSH, CAT, SOD, GPX) in Nano-HA and Nano-FA treated groups compared to the MTA group (*p* ≥ 0.05). However, it was noticed that there was an insignificant increase of NO in the Nano-FA treated group compared to both MTA and Nano-HA treated group, which may be revealed as slight vasodilation in the Nano-FA group compared to the other groups (Tables 2 and 3). *p* ≤ 0.05.

**Table 2:** Comparison of Oxidative stress markers, antioxidant enzymes, Proinflammatory cytokines, and interleukins in Serum between the three groups at 4 weeks

	**MTA (*n* = 12)**	**Nano-FA (*n* = 12)**	**Nano-HA (*n* = 12)**	**F**	***p***
**SOD (U/ml)**					
Min. – Max	24.50 – 30.20	22.10 – 31.30	26.0 – 30.40	0.497	0.620
Mean ± SD	27.16 ± 2.36	27.14 ± 4.16	28.76 ± 1.80		
**CAT (U/ml)**					
Min. – Max	16.40 – 22.60	15.30 – 23.0	17.90 – 20.40	0.058	0.944
Mean ± SD	19.48 ± 2.45	19.24 ± 3.40	18.94 ± 1.09		
**GPx (U/ml)**					
Min. – Max	5.30 – 7.70	4.0 – 6.90	3.50 – 8.70	0.089	0.915
Mean ± SD	6.04 ± 0.95	5.68 ± 1.30	5.98 ± 1.91		
**Nitric Oxide (µmol/L)**					
Min. – Max	5.20 – 6.60	5.90 – 8.90	4.20 – 6.80	2.700	0.108
Mean ± SD	5.96 ± 0.51	7.10 ± 1.26	5.72 ± 1.08		
**GSH (Nmol/ml)**					
Min. – Max	13.90 – 15.20	11.10 – 19.30	15.70 – 18.80	1.638	0.235
Mean ± SD	14.66 ± 0.54	16.36 ± 3.17	16.82 ± 1.23		
**TNFα (Pg/ ml)**					
Min. – Max	16.30 – 23.0	17.80 – 23.70	15.10 – 20.50	1.087	0.368
Mean ± SD	19.92 ± 2.90	21.02 ± 2.92	18.52 ± 2.17		
**IL-6 (Pg/ ml)**					
Min. – Max	3.20 – 4.90	5.70 – 6.60	2.50 – 6.50	6.136^*^	0.015^*^
Mean ± SD	3.82 ± 0.68	6.10 ± 0.36	4.70 ± 1.62		
**Sig. bet. grps**	**p**_**1**_ = 0.012^*^, p_**2**_** = 0.401, p**_**3**_** = 0.125**				

*F* F for One-way ANOVA test and pairwise comparison bet. each 2 groups were done using a post-hoc test (Tukey)

p: *p*-value for comparing between the studied groups

p_1_: *p*-value for comparing between MTA and Nano-FA

p_2_: *p*-value for comparing between MTA and Nano-HA

p_3_: *p*-value for comparing between Nano-FA and Nano-HA

^*^: Statistically significant at *p* ≤ 0.05

**Table 3:** Comparison of oxidative stress markers, antioxidant enzymes, proinflammatory cytokines, and interleukins in serum between the three groups at 6 weeks

	**MTA (*n* = 12)**	**Nano-FA (*n* = 12)**	**Nano-HA (*n* = 12f)**	**F**	***p***
**SOD (U/ml)**					
Min. – Max	25.30 – 30.80	21.30 – 33.0	26.0 – 32.10	0.212	0.812
Mean ± SD	28.46 ± 2.53	28.28 ± 4.42	29.56 ± 2.83		
**CAT (U/ml)**					
Min. – Max	15.60 – 25.20	15.30 – 23.0	15.90 – 20.40	0.019	0.981
Mean ± SD	19.10 ± 3.71	19.04 ± 3.49	18.74 ± 1.84		
**GPx (U/ml)**					
Min. – Max	5.30 – 7.60	4.0 – 6.90	3.50 – 8.70	0.392	0.684
Mean ± SD	5.92 ± 0.96	5.50 ± 1.21	6.30 ± 1.93		
**Nitric Oxide (µmol/L)**					
Min. – Max	5.20 – 6.40	5.30 – 8.90	4.20 – 7.70	1.435	0.276
Mean ± SD	5.90 ± 0.46	6.86 ± 1.44	5.72 ± 1.28		
**GSH (Nmol/ml)**					
Min. – Max	14.10 – 18.30	10.10 – 19.30	14.20 – 18.20	0.004	0.996
Mean ± SD	15.68 ± 1.71	15.76 ± 3.45	15.82 ± 1.54		
**TNFα (Pg/ ml)**					
Min. – Max	14.50 – 23.80	17.80 – 25.70	14.10 – 23.50	1.256	0.320
Mean ± SD	19.32 ± 4.06	22.22 ± 2.85	19.12 ± 3.36		
**IL-6 (Pg/ ml)**					
Min. – Max	3.20 – 4.90	5.70 – 7.0	2.50 – 5.50	8.168	0.006^*^
Mean ± SD	4.02 ± 0.64	6.18 ± 0.51	4.10 ± 1.44		
**Sig. bet. grps**	**p**_**1**_ = 0.010^*^, p_**2**_** = 0.990, p**_**3**_ = 0.013^*^				

*F* F for One-way ANOVA test and pairwise comparison bet. each 2 groups was done using a post-hoc test (Tukey)

p: *p*-value for comparing between the studied groups

p_1_: *p*-value for comparing between MTA and Nano-FA

p_2_: *p*-value for comparing between MTA and Nano-HA

p_3_: *p*-value for comparing between Nano-FA and Nano-HA

^*^: Statistically significant at *p* ≤ 0.05

### Proinflammatory cytokines and interleukins

At 4- and 6-week intervals, there was no significant increase or decrease in the values of TNF-α in Nano-FA and Nano-HA treated groups compared to the MTA group (*p* > 0.05.However, it was noticed that there was a significant increase (*p* < 0.05) in values of IL-6 in the Nano-FA treated group compared to MTA (at p_1_ = 0.012 and p1 = 0.010) at the two intervals**.** Comparing the Nano-FA with Nano-HA was insignificant at 4 weeks (at p3 = 0.125) and significant (*p* < 0.05) at 6 weeks (at *p*3 = 0.013), which may be manifested as some nonpathological inflammations in the Nano-FA group compared to the other groups (Table 2 and 3).

### Histomorphological results

#### Hematoxylin and Eosin stain

**Fig. 9:**
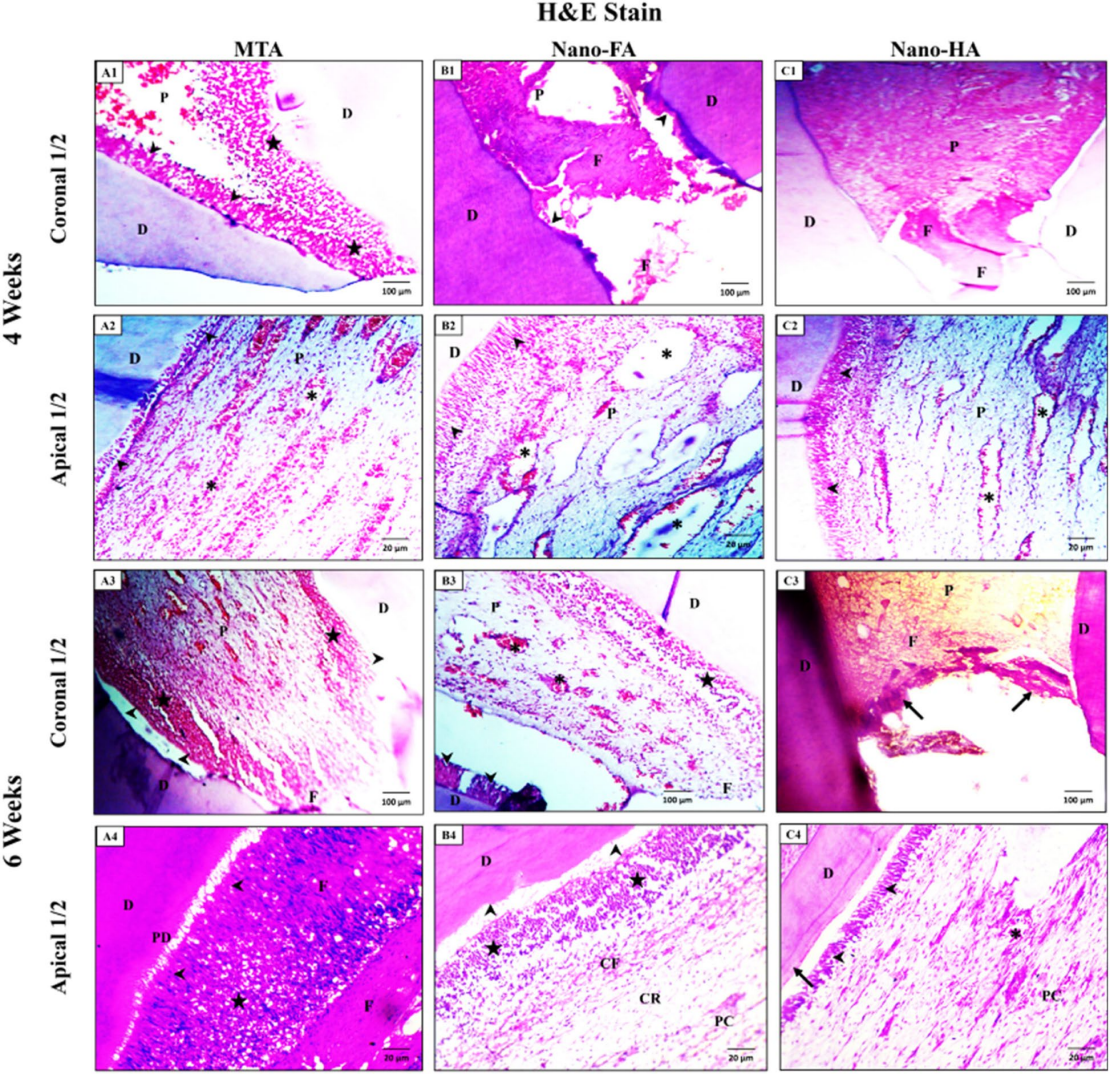
Photomicrographs of the upper incisor teeth of all groups. (A1 & A2) showing the pulp tissue after 4 weeks of MTA pulpotomy treatment. (A1) at the coronal half of the tooth with granulation tissues (stars) inside the pulp (P) at the operating site and on one side of the dentin surface (D), and a nearly intact odontoblastic layer (arrowheads) on the other side of the dentin surface. (A2) The apical half shows the pulp (P) engorged with medium-sized dilated blood vessels (*) in all pulp zones. The odontoblastic layer (arrowheads) appears intact and low columnar bordering normal dentin (D). (A3 & A4) showing the pulp tissue after 6 weeks from MTA pulpotomy treatment. (A3) The coronal half shows fibrosis (F) at the operating site, granulation tissues (stars) on two sides of the dentin surface (D), and a loss of odontoblastic layer (arrowheads) on both sides. (A4) The apical area shows a nearly normal odontoblastic layer (arrowheads). Still, it is difficult to differentiate due to a large amount of pulp fibrosis (F) and granulation tissues (stars) filling the pulp space. Notice, the normal appearance of the pre-dentine layer (PD) at the pulpal side of dentin (D). (B1 & B2) showing the pulp tissue after 4 weeks from Nano-FA pulpotomy treatment. (B1) The coronal pulp (P) shows pulp fibrosis (F) at the operating site and partial loss of the odontoblastic layer (arrowheads) at smaller areas. (B2) The apical half of the pulp (P) appears normal but engorged with many large, dilated blood vessels (*), and the odontoblastic layer appears intact, well-organized, and tall columnar all over the section (arrowheads). (B3 & B4) showing the pulp tissue after 6 weeks from Nano-FA pulpotomy treatment. (B3) The coronal area of the pulp (P) shows an intact odontoblastic layer (arrowheads) on one side of the dentin surface (D). Also, the odontoblastic layer is replaced by granulation tissues (stars) on the other side of the dentine surface (D). The increased number of blood vessels (*) is detected. (B4) The apical half of the pulp shows loss of the odontoblastic layer (arrowheads), leaving a space that borders the pulpal surface of dentin (D) and obvious granulation tissues (stars). Notice the normal architecture of cell-free (CF), cell-rich (CR), and pulp core (PC) zones. (C1 & C2) showing the pulp tissue after 4 weeks from Nano-HA pulpotomy treatment. (C1) The coronal pulp appears normal except for the fibrosis (F) at the operating site with normal dentin (D). (C2) The apical pulp (P) appears normal and engorged with medium-sized dilated blood vessels (*) in the pulp core. The odontoblastic layer appears intact, well-organized, tall columnar all over the section (arrowheads) with normal dentin (D). (C3 & C4) showing the pulp tissue after 6 weeks from Nano-HA pulpotomy treatment. (C3) The coronal pulp (P) appears intact with slight pulp fibrosis (F) under the continuous dentine bridge (arrows) at the operating site extending from the dentin surface (D) on each side. (C4) The apical half of the pulp (P) appears normal and shows a normal odontoblastic layer (arrowheads), and the pulp core (PC) depicts normal blood vessels (*). Notice the normal appearance of the pre-dentine layer (arrow). The space between the odontoblasts (arrowheads) and the dentine (D) is due to the processing technique. (H& E stain, A1, A3, B1, B3, C1 & C3 X 100 and A2, A4, B2, B4, C2 & C4 X 400)

*At 4 weeks:* the pulp tissue of the MTA group at the coronal half of the teeth showed granulation tissues at the operating site and a nearly intact odontoblastic layer on only one side of the dentin. (Fig. 9-A1). However, in the Nano-FA and Nano-HA groups, the coronal area showed pulp fibrosis at the operating site and partial loss of the odontoblastic layer. (Fig. 9-B1 & C1). The apical pulp in all three groups appeared normal and engorged with medium-sized dilated blood vessels in the pulp core and intact, well-organized, and columnar odontoblasts (Fig. 9-A2, B2 & C2).

*At 6 weeks:* The coronal half of the teeth in the MTA group showed fibrosis at the operating site, granulation tissues on two sides of the dentin surface, and complete loss of the odontoblastic layer on both sides (Fig. 9-A3). The apical area shows a nearly normal odontoblastic layer, but it is difficult to differentiate due to the presence of a large amount of pulp fibrosis and granulation tissues with a normal appearance of the pre-dentine layer (Fig. 9-A4). In the Nano-FA group, the coronal area showed an intact odontoblastic layer on one side of the dentin surface. Also, there is a replacement of the odontoblastic layer by granulation tissues on the other side. The increased number of blood vessels is detected (Fig. 9-B3). In the Nano-HA group, the coronal pulp appeared intact with slight pulp fibrosis under the continuous dentine bridge at the operating site (Fig. 9-C3). The apical half of the pulp in the Nano-FA and Nano-HA groups appeared normal and showed normal pulp layers and the pulp core depicted normal blood vessels with the normal appearance of the pre-dentine layer (Fig. 9-B4 & C4).

### Masson trichrome stains

**Fig. 10:**
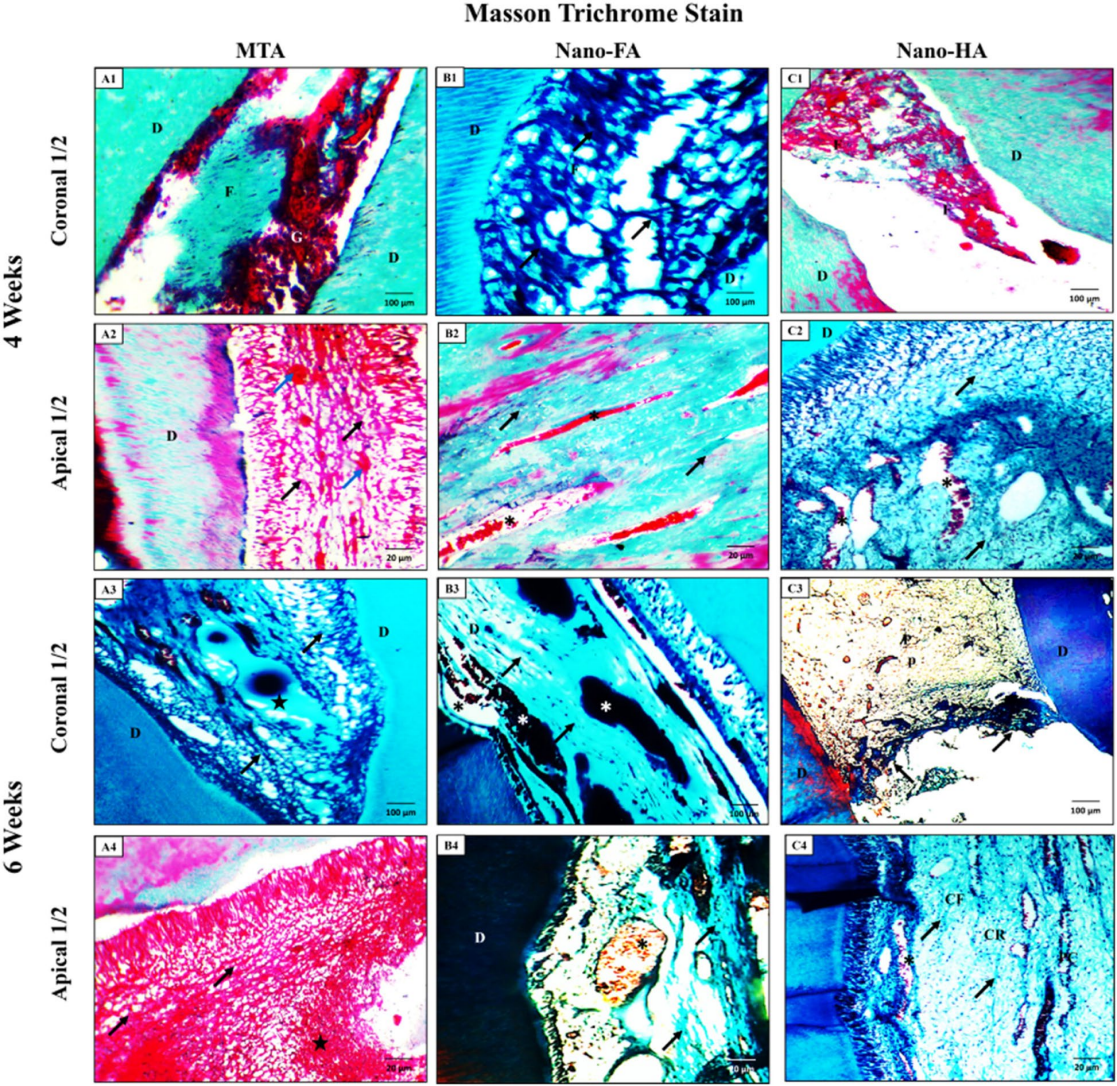
Photomicrographs of the upper incisor teeth of all groups. (A1 & A2) showing the pulp tissue, after 4 weeks from MTA pulpotomy treatment. (A1) The coronal half of the tooth appeared filled with condensed fibrous tissues (F) and granulation tissues (G). (A2) The apical half shows coarse collagen fibers (black arrows) and extravasated RBCs (blue arrows). (A3 & A4) showing the pulp tissue, after 6 weeks from MTA pulpotomy treatment. (A3) The coronal half shows coarse collagen fibers (black arrows) and a calcified area (star). (A4) The apical area shows coarse collagen Fibers (black arrows) and granulation tissues (star). (B1 & B2) showing the pulp tissue, after 4 weeks from Nano-FA pulpotomy treatment. (B1) coronal area shows pulp fibrosis (F) with obvious coarse collagen fibers (black arrows). (B2) The apical half depicts condensed collagen fibers (black arrows) and large blood vessels with extravasated RBCs (*). (B3 & B4) showing the pulp tissue, after 6 weeks from Nano-FA pulpotomy treatment. (B3) The coronal area shows condensed collagen fibers (black arrows) and large blood vessels with extravasated RBCs (*). (B4) The apical half shows medium-sized collagen fibers (black arrows) and large blood vessels with extravasated RBCs (*). (C1 & C2) showing the pulp tissue, after 4 weeks from Nano-HA pulpotomy treatment. (C1) The coronal pulp appears filled with fibrous tissue (F) at the operating site. (C2) The apical pulp (P) appears with medium-sized fibers (black arrow) and medium-sized blood vessels (*). (C3 & C4) showing the pulp tissue, after 6 weeks from Nano-HA pulpotomy treatment. (C3) The coronal pulp (P) appears intact with slight pulp fibrosis (F) under the continuous dentine bridge (arrows) at the operating site. (C4) The apical half of the pulp appears normal and shows normal pulp zones; odontoblasts, cell-free (CF), cell-rich (CR), and pulp core (PC) depict normal blood vessels (*). (Masson Trichrome stain, A1, A3, B1, B3, C1 & C3 X 100 and A2, A4, B2, B4, C2 & C4 X 400)

*At 4 weeks:* the pulp tissue at the coronal half of the teeth appeared filled with condensed fibrous tissues and granulation tissues in the MTA group (Fig. 10-A1). However, the Nano-FA and Nano-HA groups, showed pulp fibrosis at the operating site (Fig. 10-B1 & C1). Regarding the apical half, it depicted coarse collagen fibers. It extravasated red blood cells (RBCs) in the MTA group (Fig. 10-A2), many large, dilated blood vessels in the Nano-FA group (Fig. 10-B2), and medium-sized fibers and medium-sized blood vessels in the Nano-HA group (Fig. 10-C2).

*At 6 weeks:* the pulp tissue at the coronal half of the teeth showed coarse collagen fibers and a calcified area in the MTA group (Fig. 10-A3). There was a replacement of the odontoblastic layer by granulation tissues on one side of the dentin with an increased number of blood vessels in the Nano-FA group (Fig. 10-B3). Also, a slight pulp fibrosis under the continuous dentine bridge at the operating site was detected in the Nano-HA group (Fig. 10-C3). Regarding the apical half, it showed coarse collagen fibers and granulation tissues in the MTA group (Fig. 10-A4). The most obvious feature in the Nano-FA group was the large blood vessels with extravasated RBCs (Fig. 10-B4). Conversely, it appeared normal and showed normal pulp zones and normal blood vessels in the Nano-HA group (Fig. 10-C4).

## Discussion

The famous Chicago school architect Louis Sullivan first proposed that "form follows function" more than a century ago. One excellent illustration of this idea is seen in nature. Dental tissues are mineralized tissues, where HA is the main component, which is very appropriate in developments, where biomimetic Nano-HA can modulate enhanced osteoblast adhesion without causing severe pulp inflammatory response [36]. Controlling the size and shape of apatite crystals is one of the strategies that allows for adjusting the functional needs. In general, temperature, concentration of reactants, and pH of solution are critical parameters in controlling the morphology of HA particles. HA nanorods fabricated in this way are expected to act as a good candidate to mimic natural tissues. The histomorphological data generated by this study regarding the formed dentin bridge showed that Nano-HA was able to stimulate dentin bridge formation, this may be related to the calcium /phosphate ratio present in the Nano-HA which is close to the theoretical value for natural apatite; additionally, that ratio might have played a role in cell metabolic process. According to the results obtained from the X-ray diffraction investigation, the mean crystal size of the prepared Nano-HA particles was found to be 20.4 nm which was very close to that observed in human teeth [37]. Such findings support that using the same size of natural Nano-HA during dental application provides a novel pathway to repair teeth and it may also stimulate the differentiation of stem cells into fibroblast and odontoblast-like cells to promote tissue mineralization and reparative dentin [36]. Similar results were highlighted by Li et al., 2019. His work provided evidence that nanoparticles with size 20 were the most effective at promoting odontoblastic cell growth [38]. These results were also consistent with the histological results of earlier studies [39] where Nano-HA was used as a pulpotomy and dental pulp capping agent in pig teeth. The current research observed that Nano-HA showed the least inflammatory cell infiltration and persistence of normal pulpal tissue organization along with hard tissue formation. These findings support their use as pulpotomy materials over the primary pulp and were in harmony with the findings of Abd El-Azim et al., 2023 [39] who reported that when Nano-HA was used as a pulpotomy material, it had improved clinical and radiographic outcomes.

The study's findings have demonstrated that there was neither a significant increase nor decrease in oxidative stress markers and antioxidants (NO, SOD, CAT, GSH, GPX) in both Nano-FA and Nano-HA while considering inflammatory markers such as TNF-α and IL-6 measured in this study, there were also no signs of significant increase or decrease in those parameters in either Nano-FA or Nano-HA treated groups, but again there was an insignificant increase in Nano-FA treated group when compared to the other groups. These findings were in line with a recent study [40] that prepared six kinds of hydroxyapatite nanoparticles selectively that caused a reduction of osteosarcoma in vitro and in vivo in mice with no apparent systemic toxicity such as inflammation or elevated oxidative stress. Therefore, determining the antitumor activity of these particles. Nevertheless, the dose of Nano-HA must be carefully monitored, where in some research a high dose of Nano-HA may cause toxicity and elevation in oxidative stress and inflammatory markers in other organs such as the heart, liver, or kidney [41, 42]. Also, Zhao et al., 2011 [43] demonstrated that Nano-HA with smaller specific surface areas induced minimal pathological effects. However, HA with a higher surface area increased the cell-particle interaction and elevated the ROS generation, leading to oxidative stress damage and a high risk of inflammation. Furthermore, another study [44] identified that Nano-HA possessed anti-inflammatory and antimicrobial activities.

According to our knowledge, this is one of the novel types of research to test Nano-FA on dental pulp healing, where there is barely any reported clinical evidence on its effect on pulp healing. Referring to other studies [45, 46] stated that Nano-FA is considered to be antimicrobial and anti-inflammatory, and could be considered as a remineralizing agent in oral care cosmetics but their study was based on applying it to tooth enamel, not pulp. Therefore, we recommend that further studies should be done on Nano-FA to investigate their effect on pulp with more specific analysis measuring oxidative stress, inflammatory, and apoptotic markers in pulp tissue in different doses and different ranges of time intervals that could manifest their effect on dental pulp.

Histological results were consistent with the blood examination results. After 4 weeks, the Nano-FA and Nano-HA groups showed pulp fibrosis at the operating site, but the MTA showed only granulation tissues. Plus, dilated blood vessels appeared in the Nano-FA group. After 6 weeks, MTA and Nano-FA groups showed pulp fibrosis at the operating site with the persistence of dilated blood vessels with Nano-FA. The Nano-HA group showed dentin bridge formation at the operating site. The inflammation provoked by FA was denied in a recent study that studied the application of FA on the bone scaffold and found that it favored osteogenesis with nearly no inflammation [47]. In addition, the fluoride in the FA is thought to inhibit bacterial growth, thus making it compatible with dental practice [48]. However, Suzuki et al., 2015 found that FA accessible the same inflammatory response and biocompatibility as MTA [49]. The nonpathological inflammatory cell reactions, which are considered acceptable for pulp capping, produced by Nano-FA might be due to unknown reactions provoked between the incorporated material in its synthesis or with the pulp tissues that need to be evaluated and interpreted in future research.

The study's limitations include the use of rabbit teeth, which differ from human teeth in anatomy and biology, potentially limiting the direct applicability of findings to human clinical scenarios. Additionally, the short follow-up period (4–6 weeks) does not assess the long-term effects of the pulp capping materials, which restricts understanding of their sustained performance and safety. The sample size, while consisting of 24 teeth per group, may still be too small to detect subtle differences, affecting the generalizability of the results. Moreover, the controlled laboratory setting may not reflect the complexities of real-life clinical situations. Finally, the lack of a negative control group makes it challenging to determine whether the observed healing or inflammation is due to the materials or natural processes."

## Conclusion

MTA and Nano-HA could be considered favorable materials for direct pulp capping, while Nano-FA produces nonpathological inflammatory cell reactions. Moreover, the Nano-HA was the best in dentin bridge formation. Although nano-FA increased the operating site closure, it was noticed that it significantly increased IL-6 compared to MTA at the two intervals and significantly increased IL-6 compared to Nano-HA at 6 weeks, which may be manifested as some nonpathological inflammations in the Nano-FA group compared to the other groups but it was deemed acceptable to direct pulp capping procedures.

## Data Availability

Available on request from the corresponding author.

## Abbreviations

LM: Light micrographs
MTA: Mineral trioxide aggregate
Nano-FA: Nanoparticles of fluorapatite
Nano-HA: Nanoparticles of hydroxyapatite
NO: Antioxidant enzymes nitric oxide
SOD: Superoxide dismutase
CAT: Catalase
GPx: Glutathione peroxidase
GSH: Glutathione
IL-6: Interleukin-6
TNF-α: Tumor necrosis factor-α
DPC: Direct pulp capping

## References

[1] Pashley DH. Dynamics of the pulpo-dentin complex. Crit Rev Oral Biol Med. 1996;7(2):104–33. 10.1177/10454411960070020101. 8875027 10.1177/10454411960070020101

[2] Hatipoğlu Ö, Hatipoğlu FP, Javed MQ, Nijakowski K, Taha N, El-Saaidi C, et al. Factors affecting the decision-making of direct pulp capping procedures among dental practitioners: a multinational survey from 16 countries with meta-analysis. J Endod. 2023;49:675–85. 37094712 10.1016/j.joen.2023.04.005

[3] Su Y, Wang C, Ye L. Healing rate and post-obturation pain of single- versus multiple-visit endodontic treatment for infected root canals: a systematic review. J Endod. 2011;37(2):125–32. 10.1016/j.joen.2010.09.005. 21238790 10.1016/j.joen.2010.09.005

[4] Duncan HF. Present status and future directions-Vital pulp treatment and pulp preservation strategies. Int Endod J. 2022;55 Suppl 3(Suppl 3):497–511. 10.1111/iej.13688. 35080024 10.1111/iej.13688 PMC9306596

[5] Altaii M, Richards L, Rossi-Fedele G. Histological assessment of regenerative endodontic treatment in animal studies with different scaffolds: a systematic review. Dent Traumatol. 2017;33(4):235–44. 10.1111/edt.12338. 28342218 10.1111/edt.12338

[6] Cao Y, Song M, Kim E, Shon W, Chugal N, Bogen G, et al. Pulp-dentin regeneration: current state and future prospects. J Dent Res. 2015;94(11):1544–51. 10.1177/0022034515601658. 26310721 10.1177/0022034515601658

[7] Nakashima M, Iohara K. Regeneration of dental pulp by stem cells. Adv Dent Res. 2011;23(3):313–9. 10.1177/0022034511405323. 21677085 10.1177/0022034511405323

[8] Modena KC, Casas-Apayco LC, Atta MT, Costa CA, Hebling J, Sipert CR, et al. Cytotoxicity and biocompatibility of direct and indirect pulp capping materials. J Appl Oral Sci. 2009;17(6):544–54. 10.1590/s1678-77572009000600002. 20027424 10.1590/S1678-77572009000600002 PMC4327511

[9] Hilton TJ. Keys to clinical success with pulp capping: a review of the literature. Oper Dent. 2009;34(5):615–25. 10.2341/09-132-0. 19830978 10.2341/09-132-0 PMC2856472

[10] Khorakian F, Mazhari F, Asgary S, Sahebnasagh M, AlizadehKaseb A, Movahhed T, et al. Two-year outcomes of electrosurgery and calcium-enriched mixture pulpotomy in primary teeth: a randomised clinical trial. Eur Arch Paediatr Dent. 2014;15(4):223–8. 10.1007/s40368-013-0102-z. 24435546 10.1007/s40368-013-0102-z

[11] Fuks A, Kupietzky A, Guelmann M. Pulp therapy for the primary dentition. In: Nowak A, Christensen J, Mabry T, Townsend J, Wells M, editors. Pediatric DentistryInfancy through Adolescence. 6th ed. St. Louis, Mo: Elsevier-Saunders Co; 2019. p. 329–51.

[12] Alex G. Direct and indirect pulp capping: a brief history, material innovations, and clinical case report. Compend Contin Educ Dent. 2018;39(3):182–9. 29493248

[13] Mente J, Hufnagel S, Leo M, Michel A, Gehrig H, Panagidis D, et al. Treatment outcome of mineral trioxide aggregate or calcium hydroxide direct pulp capping: long-term results. J Endod. 2014;40(11):1746–51. 10.1016/j.joen.2014.07.019. 25227216 10.1016/j.joen.2014.07.019

[14] Stanley HR, Clark AE, Pameijer CH, Louw NP. Pulp capping with a modified bioglass formula (#A68-modified). Am J Dent. 2001;14(4):227–32. 11699742

[15] Zanini M, Sautier JM, Berdal A, Simon S. Biodentine induces immortalized murine pulp cell differentiation into odontoblast-like cells and stimulates biomineralization. J Endod. 2012;38(9):1220–6. 10.1016/j.joen.2012.04.018. 22892739 10.1016/j.joen.2012.04.018

[16] Mitra SB, Wu D, Holmes BN. An application of nanotechnology in advanced dental materials. J Am Dent Assoc. 2003;134(10):1382–90. 10.14219/jada.archive.2003.0054. 14620019 10.14219/jada.archive.2003.0054

[17] Qureshi A, E S, Nandakumar, Pratapkumar, Sambashivarao. Recent advances in pulp capping materials: an overview. J Clin Diagn Res. 2014;8(1):316–21. 10.7860/jcdr/2014/7719.3980. 10.7860/JCDR/2014/7719.3980 PMC3939574 24596805

[18] Swarup SJ, Rao A, Boaz K, Srikant N, Shenoy R. Pulpal response to nano hydroxyapatite, mineral trioxide aggregate and calcium hydroxide when used as a direct pulp capping agent: an in vivo study. J Clin Pediatr Dent. 2014;38(3):201–6. 10.17796/jcpd.38.3.83121661121g6773. 25095313 10.17796/jcpd.38.3.83121661121g6773

[19] Boone ME 2nd, Kafrawy AH. Pulp reaction to a tricalcium phosphate ceramic capping agent. Oral Surg Oral Med Oral Pathol. 1979;47(4):369–71. 10.1016/0030-4220(79)90262-7. 107502 10.1016/0030-4220(79)90262-7

[20] Kantharia N, Naik S, Apte S, Kheur M, Kheur S, Kale B. Nano-hydroxyapatite and its contemporary applications. J Dent Res Scic Dev. 2014;1(1):15–9.

[21] Bedir MA, Amin L. Potential effect of different pulp capping materials on human dental pulp stem cells: a laboratory study. Mansoura J Dent. 2023;10(2):88–96.

[22] Kim HW, Kim HE, Knowles JC. Fluor-hydroxyapatite sol-gel coating on titanium substrate for hard tissue implants. Biomaterials. 2004;25(17):3351–8. 10.1016/j.biomaterials.2003.09.104. 15020107 10.1016/j.biomaterials.2003.09.104

[23] Ashall V, Morton D, Clutton E. A Declaration of Helsinki for animals. Vet Anaesth Analg. 2023;50(4):309–14. 10.1016/j.vaa.2023.03.005. 37183079 10.1016/j.vaa.2023.03.005

[24] Tredwin CJ, Young AM, Abou Neel EA, Georgiou G, Knowles JC. Hydroxyapatite, fluor-hydroxyapatite and fluorapatite produced via the sol-gel method: dissolution behaviour and biological properties after crystallisation. J Mater Sci Mater Med. 2014;25(1):47–53. 10.1007/s10856-013-5050-y. 24052344 10.1007/s10856-013-5050-y PMC3890558

[25] Wang X, Jin T, Chang S, Zhang Z, Czajka-Jakubowska A, Nör JE, et al. In vitro differentiation and mineralization of dental pulp stem cells on enamel-like fluorapatite surfaces. Tissue Eng Part C Methods. 2012;18(11):821–30. 10.1089/ten.TEC.2011.0624. 22563788 10.1089/ten.tec.2011.0624 PMC3483051

[26] Charan J, Biswas T. How to calculate sample size for different study designs in medical research? Indian J Psychol Med. 2013;35(2):121–6. 10.4103/0253-7176.116232. 24049221 10.4103/0253-7176.116232 PMC3775042

[27] Pannucci CJ, Wilkins EG. Identifying and avoiding bias in research. Plast Reconstr Surg. 2010;126(2):619–25. 10.1097/PRS.0b013e3181de24bc. 20679844 10.1097/PRS.0b013e3181de24bc PMC2917255

[28] Moayedee Y, Mobasherpour I, Banijamali S, Razavi M, Nezafati N. Effect of the nano-fluorapatite ceramic particles on mechanical behavior of fluoride varnishes. Materials Chem Phys. 2022;288:126421. 10.1016/j.matchemphys.2022.126421.

[29] Bianco A, Cacciotti I, Lombardi M, Montanaro L, Gusmano G. Thermalstability and sintering behaviour of hydroxyapatite nanopowders. J Therm Anal Calorim. 2007;88(1):237–43. 10.1007/s10973-006-8011-6.

[30] Patterson A. The Scherrer formula for X-Ray particle size determination". Phys Rev. 1939;56(10):978–82.

[31] Pichaiaukrit W, Thamrongananskul N, Siralertmukul K, Swasdison S. Fluoride varnish containing chitosan demonstrated sustained fluoride release. Dent Mater J. 2019;38(6):1036–42. 10.4012/dmj.2018-112. 31611494 10.4012/dmj.2018-112

[32] Wei M, Evans JH, Bostrom T, Grøndahl L. Synthesis and characterization of hydroxyapatite, fluoride-substituted hydroxyapatite and fluorapatite. J Mater Sci Mater Med. 2003;14(4):311–20. 10.1023/a:1022975730730. 15348455 10.1023/a:1022975730730

[33] Montazeri N, Jahandideh R, Biazar E. Synthesis of fluorapatite-hydroxyapatite nanoparticles and toxicity investigations. Int J Nanomedicine. 2011;6:197–201. 10.2147/ijn.s15461. 21499417 10.2147/IJN.S15461 PMC3075893

[34] Herman K, Wujczyk M. In Vitro assessment of long-term fluoride ion release from nanofluorapatite. Materials (Basel). 2021;14(13):3747. 10.3390/ma14133747. 34279317 10.3390/ma14133747 PMC8269907

[35] Jean A, Kerebel B, Kerebel LM, Legeros RZ, Hamel H. Effects of various calcium phosphate biomaterials on reparative dentin bridge formation. J Endod. 1988;14(2):83–7. 10.1016/s0099-2399(88)80006-2. 3162944 10.1016/S0099-2399(88)80006-2

[36] Li L, Pan H, Tao J, Xu X, Mao C, Gu X, et al. Repair of enamel by using hydroxyapatite nanoparticles as the building blocks. J Mat Chem. 2008;18(34):4079–84.

[37] Li N, Wu G, Yao H, Tang R, Gu X, Tu C. Size effect of nano-hydroxyapatite on proliferation of odontoblast-like MDPC-23 cells. Dent Mater J. 2019;38(4):534–9. 10.4012/dmj.2018-155. 30787214 10.4012/dmj.2018-155

[38] Shayegan A, Atash R, Petein M, Abbeele AV. Nanohydroxyapatite used as a pulpotomy and direct pulp capping agent in primary pig teeth. J Dent Child (Chic). 2010;77(2):77–83. 20819402

[39] Abd El-Azim S, Baiomy S, Barakat I. Clinical and radiographic evaluation of nanohydroxyapatite and platelets-rich fibrin as pulpotomy materials for primary molars. Int J Adv Res. 2023;11:114–20. 10.21474/IJAR01/15997.

[40] Wu H, Liu S, Chen S, Hua Y, Li X, Zeng Q, et al. A selective reduction of osteosarcoma by mitochondrial apoptosis using hydroxyapatite nanoparticles. Int J Nanomedicine. 2022;17:3691–710. 10.2147/ijn.s375950. 36046839 10.2147/IJN.S375950 PMC9423115

[41] El-Masry T, Altwaijry N, Alotaibi B, Tousson E, Alboghdadly A, Saleh A. Chicory (Cichorium intybus L.) extract ameliorates hydroxyapatite nanoparticles induced kidney damage in rats. Pak J Pharm Sci. 2020;33(3):1251–60.

[42] Mosa IF, Abd HH, Abuzreda A, Yousif AB, Assaf N. Chitosan and curcumin nanoformulations against potential cardiac risks associated with hydroxyapatite nanoparticles in wistar male rats. Int J Biomater. 2021;2021:3394348. 10.1155/2021/3394348. 34373695 10.1155/2021/3394348 PMC8349268

[43] Zhao X, Heng BC, Xiong S, Guo J, Tan TT, Boey FY, et al. In vitro assessment of cellular responses to rod-shaped hydroxyapatite nanoparticles of varying lengths and surface areas. Nanotoxicology. 2011;5(2):182–94. 10.3109/17435390.2010.503943. 21609137 10.3109/17435390.2010.503943

[44] Martínez-Sanmiguel JJ, D GZ-T, Hernandez-Delgadillo R, Giraldo-Betancur AL, Pineda-Aguilar N, Galindo-Rodríguez SA, et al. Anti-inflammatory and antimicrobial activity of bioactive hydroxyapatite/silver nanocomposites. J Biomater Appl. 2019;33(10):1314–26. 10.1177/0885328219835995 10.1177/0885328219835995 30880564

[45] Carrouel F, Viennot S, Ottolenghi L, Gaillard C, Bourgeois D. Nanoparticles as anti-microbial, anti-inflammatory, and remineralizing agents in oral care cosmetics: a review of the current situation. Nanomaterials (Basel). 2020;10(1):140. 10.3390/nano10010140. 31941021 10.3390/nano10010140 PMC7022934

[46] Gronwald B, Kozłowska L, Kijak K, Lietz-Kijak D, Skomro P, Gronwald K, et al. Nanoparticles in dentistry—current literature review. Coatings. 2023;13(1):102. 10.3390/coatings13010102.

[47] Seyedmajidi S, Seyedmajidi M, Haghanifar S. Optimization of fluorapatite/bioactive glass nanocomposite foams as bone tissue scaffold: an in vivo study. Int J Mol Cell Med. 2023;12(4):388–400. 10.22088/ijmcm.bums.12.4.388. 39006199 10.22088/IJMCM.BUMS.12.4.388 PMC11240056

[48] Kanchana P, Sekar C. Influence of sodium fluoride on the synthesis of hydroxyapatite by gel method. J Cryst Growth. 2010;312(6):808–16.

[49] Suzuki Y, Hayashi M, Yasukawa T, Kobayashi H, Makino K, Hirano Y, et al. Development of a novel fluorapatite-forming calcium phosphate cement with calcium silicate: in vitro and in vivo characteristics. Dent Mater J. 2015;34:263–9. 25740309 10.4012/dmj.2014-255

